# Identifying Hate Speech and Attribution of Responsibility: An Analysis of Simulated WhatsApp Conversations during the Pandemic

**DOI:** 10.3390/healthcare11111564

**Published:** 2023-05-26

**Authors:** José Luis Palazón-Fernández, Concepción Mata-Perez, Ester Gilart, Eva Manuela Cotobal Calvo, Alberto Cruz-Barrientos, Anna Bocchino

**Affiliations:** 1 Nursing University Salus Infirmorum, 11001 Cádiz, Spain; jluis.palazon@uca.es (J.L.P.-F.); conchi.mata@ca.uca.es (C.M.-P.); evamanuela.cotobal@ca.uca.es (E.M.C.C.); alberto.cruzba@ca.uca.es (A.C.-B.); 2 Department of Biomedicine, Biotechnology and Public Health, University of Cadiz, 11001 Cádiz, Spain; ester.gilart@uca.es

**Keywords:** COVID-19 vaccine, hate speech, responsibility, perceived aggressiveness

## Abstract

Background: During the COVID-19 pandemic, public confrontations between people who had agreed to be vaccinated and those who had not, highlighted the relevance of the deepening dissemination of violent and discriminatory expressions and determined a level of perception of hate discourses. Method: A cross-sectional observational study was carried out, based on an innovative methodology: simulations of WhatsApp conversations. In addition, the following variables were considered among others: level of empathy, personality traits and conflict resolution. Results: The participants were 567 nursing students (413 females, 153 males and 1 person who did not identify with any gender). The results showed that, for the most part, the participants correctly identified hate speech, but were unable to discern the frame of reference. Conclusions: It is necessary to implement intervention strategies to minimize the impact of hate speech, which continues to be used on many levels to harass others, justify violence or undermine rights, generating an environment of prejudice and intolerance that encourages discrimination and violent attacks against certain individuals or collectives.

## 1. Introduction

The issue of hate speech often sparks controversy since, to date, there is no unanimous or universally accepted definition for it [1]. Being highly subjective, it is a concept that creates a great deal of confusion [2]. There does seem to be consensus, however, when considering hate speech, that it is the transmission of verbal or written pejorative expressions that directly incite discriminatory acts towards a person or a collective [3,4].

Hate speech comes in many guises and its repercussions are varied. On the one hand, malicious messages, harassment and other types of threatening messages can cause emotional or psychological harm to the recipients. On the other hand, the indirect consequences caused by the continued legitimization and proliferation of such speech can generate passivity among bystanders or observers in the face of the perpetuation of discriminatory stereotypes and the stigmatization of certain groups. Thus, “there is a reduction of empathy towards dehumanized collectives generating the right breeding ground to justify discriminatory acts, abuse and violent acts of various nature” [2].

Moreover, the approval of hate speech among peer groups, for example, facilitates the reinforcement of the issuer’s feeling of belonging to the aggressor group [5]. These discourses are further reinforced and deepened in the digital context [6,7]. In fact, on some occasions, information and communication technology (ICT) and social media applications have become contexts of dispute, fragmentation, polarization and extremism [8]. An exemplification of this phenomenon is provided by the WhatsApp application, which affords a platform for both individual and collective dialogues, seeking to establish a singular framework for interpreting events, as well as a correct and typically conventional modality for interpersonal engagement within the respective communities. Nevertheless, the excessive utilization of this application can also render it a conducive medium for the dissemination and proliferation of hate-filled and violent messages among its users. These forms of communication, referred to as “hate speech”, primarily target vulnerable or disparate groups perceived as threats or culpable for disrupting the political and social order.

Therefore, hate speech can silence certain vulnerable social groups, preventing them from acting freely in their daily lives or in the democratic forum, thus becoming an impediment to freedom of expression [9]. Moreover, it can aggravate its destructive potential at an emotional, personal or collective level, affecting the dignity of the people against whom such manifestations are directed [10,11]. Moreover, on some occasions and under some circumstances, such hate speech can contribute to trigger violence [2,12]. The concern regarding the potential incitement of violence through this type of messaging gives rise to a call for political, social, and legal responses. Specifically, it raises the question of why adolescents are among the primary agents involved in transmitting these discourses. Despite the scarcity of studies analyzing the underlying motivations behind such speech, as well as the limited research on how young individuals perceive or detect hate speech, it is indisputable that accurately identifying violent speech could serve as a catalyst for preventing its dissemination in both public and private spheres. This identification would undoubtedly prove beneficial in addressing the proliferation of this type of speech in the “new contexts” they continue to emerge in.

During the past year, significant dissemination of extremist messages related to the COVID-19 vaccine has occurred throughout Europe. The topic of mandatory vaccination to safeguard against and prevent SARS-CoV-2 infection has been at the forefront of extensive discussions involving not only scientific and legal investigations but also various sectors of the general public and private sphere. An alarming rise in the circulation of hate speech targeting individuals who have received the vaccine or are inclined to receive it has been observed. This escalation has been amplified by numerous theories expressing doubts about vaccine effectiveness, particularly concerning issues related to accessing public spaces and workplaces.

Furthermore, the endorsement of conspiracy theories is associated with negative attitudes towards vaccination [13,14], which foster the dissemination of verbal or written expressions that are violent, aggressive, and discriminatory towards individuals belonging to the same social class or group. This hostility likely stems from the insecurity and misinformation surrounding all aspects of the ongoing health crisis [15]. Public confrontations between vaccinated individuals and those who are not have underscored the importance of aligning opinions to avoid the risk of victimization or discrimination, and the necessity of advancing in conceptualization and systematically acquiring effective responses to address this concerning situation.

Taking into account that discourse is a point of connection through which unequal power relations are justified, and that the propagation of violent and discriminatory expressions against vaccinated people is a highly topical issue, the present research aimed to adopt a broad approach to the issue of hate speech based on an innovative and novel methodology: simulations of WhatsApp conversations.

Specifically, the present study analyzed the participants’ perception and/or identification of violent speech (moral and physical) in both public and private contexts and the possible moderating effect of personal variables (personality, level of empathy, style of dealing with conflict).The main objective of the present study was to explore nursing students’ perception and identification of major hate speech through the attribution of the responsibility of protagonists in simulated WhatsApp conversations.

In the present research, a methodology that simulated conversations between WhatsApp users was chosen for two reasons:

1. First, an innovative methodology was followed, previously used in only one other study [16]. This can be a useful way for young people to identify violent situations or contexts;
2. Among the new technologies, WhatsApp is the only one that does not provide users with anonymity, and it is not possible to create a fictitious profile or use pseudonyms to be included in a group of friends. It was considered that this element could be fundamental when discriminating between violent and non-violent speech.

## 2. Materials and Methods

### 2.1. Participants

Using the G-Power 3.1 program, a minimum sample size of *n* = 305 was calculated for an effect size of 0.25, alpha = 0.05 and a power of 0.95. Nevertheless, an attempt was made to include as many students as possible, so the final sample consisted of a total of 567 participants (413 females, 153 males and 1 person who did not identify with any gender). The median age was 21 years, and all the subjects were studying for a degree in nursing at two universities, one public (*n* = 360) and one subsidized (*n* = 207).Most were in their second (33.0%) or third year (37.5%).

### 2.2. Instruments

Simulated WhatsApp conversations. A total of five WhatsApp conversations were used (three individual conversations and two group conversations). The conversations were between two friends (situations 1, 2 and 3) and between three friends (situations 4 and 5). In the latter two situations, the third protagonist acted as a passive observer. All scenarios begin with the same story. Protagonist 1, who is the unvaccinated person, presents the same situation in all scenarios: going to a concert despite having a severe cold. It is from this premise that the discussion between the protagonists begins, as the person in favor of vaccination considers it unnecessary to risk one’s own health and that of others to attend a concert. However, each conversation presented a different conclusion, referring to different hate speech according to the taxonomy of Llinares [10]:

1. Speech that offends or causes personal moral harm;
2. Speech that offends or causes collective moral damage;
3. Hate speech referring to physical harm against one or more persons.

The students had to read each of the scenarios and answer three questions regarding the perceived aggressiveness, the frequency of experiencing a similar situation and the level of responsibility of each of the characters in the conversations on a Likert scale from 1 to 5 (1: not at all; 5: very much).

Brief Personality Questionnaire (CBP) [17].This is an original Spanish version of a tool for assessing personality consisting of 20 items scored on a 5-point Likert-type scale (1 = completely false; 5 = completely true). It evaluates the five major personality factors: I, extroversion (items 1,6,11,16); II, agreeableness (items 2,7,12,17); III, responsibility (items 3,8,13,18); IV, neuroticism (items 4,9,14,19) and V, openness and intellect (items 5,10,15,20). Reliability, measured by Cronbach’s alpha, is between 0.61 and 0.79 for all dimensions.

Conflictalk. The self-reported survey was assessed using the original version [18] in its adaptation and validation in Spanish [19] to identify the style of conflict management in young people and adolescents. It consists of 18 items that could be expressed in a conflict situation, and the participant is asked to rate each statement on a 5-point Likert-type scale (1: I never say things like that; 5 = I almost always say things like that).

It measures three conflict resolution styles: self-focused (aggressive: implies being focused on oneself, wanting things to be done one’s own way; in the face of conflict one acts aggressively and authoritatively); problem-focused (cooperative: implies showing interest in the cause of the conflict and in specifically identifying the problem in collaboration with the other; the interest is focused on finding the best solution and acting cooperatively); and other-focused (avoidant: implies thinking that conflict is always bad, in the face of conflict one acts passively). To obtain the scores for each style, the points of the corresponding items were added: cooperative resolution (items 3, 5, 7, 11, 12, 17); avoidant (items 2, 4, 6, 13, 14, 15 and aggressive (items 1, 8, 9, 10, 16, 18). The internal consistency (Cronbach’s alpha) for the three styles was adequate (cooperative: 0.87, aggressive = 0.81, and avoidant = 0.63).

Identification with the characters. This was evaluated with the Spanish original version of EDI scale of Igartua and Paez [20]. The scale consists of 17 items (examples: “I felt as if I were one of the protagonists”, “I tried to see things from the protagonists’ point of view”), whose response format is a five-point intensity scale (1 = not at all; 5 = very much) and offers an overall indicator of identification with the characters (α = 0.88). The higher the score on the scale as a whole, the greater the identification with the characters.

### 2.3. Procedure

The cross-sectional observational study was carried out in 2022. The participants were recruited by convenience sampling. Data collection took place in the classrooms of the University Center of Nursing Salus Infirmorum of Cadiz and the Faculty of Nursing and Physiotherapy of the University of Cadiz.

The students who agreed to participate had to answer some socio-demographic questions (age, sex, faculty) and were asked to view the simulated conversations on a computer. At the end of each viewing, they were asked to rate the perceived aggressiveness, the frequency of experiencing a similar situation and the level of responsibility of each of the characters in the conversations.

Finally, after viewing the five conversations, they were asked to complete the various scales used to evaluate the independent variables of the study (personality, style of dealing with conflict, and identification with the characters).

The results of all the questionnaires were collected on-line. The approximate time required to answer all the questionnaires was 20 min.

This study was conducted in accordance with the 2013 Declaration of Helsinki (seventh revision, 64th meeting, Fortaleza) and the Organic Law 3/2018 of December 5, on the protection of personal data and guarantees of digital rights in Spain. Prior to data collection, one of the investigators explained the objectives of the study and asked all the participants to sign the informed consent. They were informed that participation was completely voluntary and at any time they could decide to leave the study if they did not feel comfortable for any reason. It was emphasized that responses were anonymous and confidential to promote honesty. To ensure the privacy of the participants, only the research team had access to the data collected.

### 2.4. Data Analysis

A preliminary analysis using the Kolmogorov–Smirnov test indicated that the data did not present a normal distribution so non-parametric statistical tests were used to perform the analyses.

The Friedman test was used to analyze the differences between the different scenarios. Tests with a *p* value ≤ 0.05 were considered significant. The Dunn–Bonferroni test was used as a post hoc test. The *p* values were corrected using the Bonferroni correction.

A correlation analysis was performed using Spearman’s coefficient to explore the possible correlations between personality styles, conflict approach style and level of identification with the characters and perceived aggressiveness, the frequency of experiencing a similar situation and the level of responsibility assigned to each of the characters in the conversations.

All the data were analyzed using the statistical package IBM SPSS version 26.0 for Windows (IBM Corp., Armonk, NY, USA).

## 3. Results

### 3.1. Personality, Conflict Resolution Style and Identification with the Characters

Table 1 presents the scores obtained for the five personality dimensions, conflict resolution styles and identification with the characters.

Regarding the personality dimensions, the traits with the highest median were “agreeableness” and “openness/intellect”, whose medians were slightly above the midpoint of the scale (12). As for the Conflictalk scale, the students referred to “cooperation” and/or “avoidance” as their preferred strategies for handling conflict situations. Finally, in relation to the results of the scale “identification with the characters”, most of the students seemed to identify well with the protagonists. That is, their level of empathy was slightly above the mean value of the scale (51).

### 3.2. Experience of a Similar Situation

The median scores for this variable were between 2 and 3 (Table 2). The results of the Friedman test indicated significant differences (*p* < 0.05) among the five simulated situations in terms of the students’ perception of having experienced a similar situation. The post hoc test indicated that the subjects considered situations 1 and 4 as the most frequently experienced, while situations 2, 3 and 5 were considered as the least similar to situations experienced in their daily life.

No correlation was observed between the experience of a similar situation and the personality traits of the participants in any of the simulated scenarios. In relation to the types of conflict management approach, most of the correlations with this variable were very low or not significant (Table 3). Those that were significant showed a direct relationship.

As for the correlations with identification with the characters, although low, these were significant in all scenarios and indicated that the more the subjects identified with the protagonists, the greater the perception of having experienced a similar situation (Table 3).

### 3.3. Perceived Aggressiveness

When analyzing how the students perceived aggressiveness according to the type of conversation, we found high scores in relation to the midpoint of the scale and significant differences (*p* < 0.05) among the five simulated scenarios. The post hoc test indicated that the conversations perceived as “less aggressive” were two and four, while conversations three and five reached the highest scores of perceived aggressiveness. No significant differences were found between scenarios one and four (Table 2).

Generally speaking, the correlations between perceived aggressiveness and personality patterns, identification with the characters and conflict resolution style, although significant in some cases (Table 3), were low in all scenarios, except for the friendliness factor, which in scenarios one and two showed a high and direct correlation (r = 0.713; *p* < 0.01 and r = 0.689; *p* < 0.01, respectively). In addition, high negative correlations were observed between perceived aggressiveness and aggressive conflict resolution style in scenarios one (r = −0.938; *p* < 0.01) and two (r = −0.874; *p* < 0.01), while in the rest, the correlations were low. Regarding identification with the characters, the correlations in the different scenarios were low or not significant.

### 3.4. Responsibility Attributed to the Characters

Regarding the results related to the levels of responsibility attributed by the students to the different characters, Friedman’s test showed significant differences (*p* < 0.05) among the five simulated scenarios (Table 2). In all five, protagonist one, i.e., the provaccine person, was considered more responsible for using hate speech than the antivaccine protagonist. Finally, in scenarios four and five the passive observer was attributed greater responsibility than the antivaccine protagonist, although always less than the provaccine protagonist.

Regarding the correlations between the responsibility attributed to each protagonist and the personality and conflict resolution styles (Table 3), the correlations were very low or not significant. In relation to “identification with the characters”, the significant correlations indicated a positive correlation with the responsibility of the provaccine protagonist and a negative correlation with the responsibility attributed to the antivaccine person. Along the same lines, a significant positive correlation was observed between identification with the characters and the attribution of responsibility to the third person (passive observer) of scenarios four and five. All of the above correlations were very low (<0.20).

### 3.5. Correlations between “Frequency of Lived Experience”, “Perceived Aggressiveness” and “Character Responsibility”

Table 4 presents the correlations between “frequency of lived experience”, “perceived aggressiveness”, and “character responsibility” in the different scenarios. All the correlations were low or very low. A negative but low correlation existed between aggressiveness and frequency of lived experience in scenarios three (r = −0.174; *p* < 0.01) and five (r = −0.102; *p* < 0.01), but not in scenarios one, two and four.

## 4. Discussion

It is unquestionable that hate speech is undergoing a consistent expansion, reaching its peak manifestation in information and communication technologies (ICT) and social networks [20,21]. Nonetheless, research on the perceived aggressiveness of such discourse remains limited [5].

The aim of the present study was to examine the experiences of students and their perception of aggressiveness and level of responsibility in different hate speech related to the COVID-19 vaccine.

Five types of hate speech expressed through simulated WhatsApp conversations were presented to the subjects.

The descriptive statistics of the sample turned out to be in line with numerous investigations according to which students of health sciences, especially medicine and nursing, stand out for their empathetic, open, extroverted and cooperative profile, probably linked to the vocational nature of their chosen profession [22].

Based on this profile, we will try to explain and discuss the results of our study.

Regarding the different scenarios, there was evidence that one and four were those that the students had the most experience of, and in fact they were not perceived as the two most aggressive scenarios, probably due, as other studies indicated, to the factors “experience or knowledge” and “third person perception” [23]. According to research in different settings [24,25,26,27], people that have shared similar experiences tend to perceive hate messages as if they were alien to them, thinking that they have a greater influence on others than on themselves. In this sense, it does not seem that our findings differ so much, especially considering that the subjects considered scenario five to be the most aggressive, which is considered to be alien to the lived experiences. Specifically, scenario five focused on one kind of hate, the narrative of which is based on a type of hate speech that offends or causes physical harm. In other words, the threat of physical harm is considered more aggressive than moral harm. This result is rather surprising in that other authors have argued that some hate speech that does not include a direct incitement/threat of physical violence but rather conveys a type of message dissemination that may cause moral harm, is perceived to be more aggressive. This is a result of the complex and controversial nature of moral harm, which may include feelings of guilt, shame, anger, disgust and sadness, thoughts of personal regret and systemic failures, and avoidance and self-handicapping behavior [28].Contrary to these findings, in our study, the results seem to suggest that the subjects were more affected by physical violence, perhaps because of its impulsive and impetuous nature. In fact, scenario two, in which the discourse is represented by moral and collective harm, is considered one of the least aggressive conversations.

Another result of note was the level of responsibility attributed to the protagonists of the speech. When considering this type of research, one of our main hypotheses was that the students’ perception of the level of responsibility would have been in favor of attributing the most responsibility to the unvaccinated person, especially considering the sample consisted of future health professionals. However, in all five scenarios, the students reported that the person responsible for the violent speech was the person who had been vaccinated, followed in cases four and five by the third person involved in the conversation, i.e., the passive observer. It is true that the vaccinated person is responsible for starting the discussion and attacking, and it is probably for this reason that they attribute more responsibility to them.

The numerous research studies into the perception of the characterizing dimensions of violence and/or hatred in a digital context could help us to gain a better understanding of this result.

For Crosslin and Golman [29], for example, the intention to harm or hurt is considered an important factor in order for adolescents to perceive an episode of aggression or cyberbullying. In our simulated conversations, the unvaccinated person does not intend to harm but to provide a defense against the vaccinated person’s attack.

Another aspect to take into account, as pointed out by other research [30,31], is the power imbalance between the aggressor and the victim. If this imbalance is evident, the more the roles of victim and aggressor will be perceived. In our case, there was probably no power inequality between the protagonists and consequently the students were not able to discern that the violent discourse was taking place within a specific context and that in this same context the role of the victim and the aggressor could change.

On the other hand, the responsibility attributed to the passive observer found in our work is in line with other studies that highlight the role of third parties, who play a decisive, participatory and relevant part in both strategies that seek to solve the problem and in the adoption of behavior that stimulates and perpetuates the cycle of violence [32,33].

Finally, it should be commented that in the results obtained from the analysis of correlations, relevant data were obtained that were congruent to other studies, among which we highlight:

- Some personality traits and/or strategies for coping with problems can influence the perception of aggressiveness [34].In fact, the trait of “kindness” is directly correlated with this perception, while an “aggressive” conflict management is inversely correlated with it.
- Identifying more with protagonists and/or empathy influence the perception of aggressiveness [35,36,37] and the attribution of the level of responsibility [38,39].

Despite the new empirical data provided by this study, we cannot ignore certain limitations. Firstly, it should be taken into account that, due to the few studies available on the perception of hate speech among a population of young people, it is difficult to conduct an exhaustive comparison of the results. Secondly, although the sample size is adequate, it should be noted that most of the subjects were Spanish and all of them were students of health sciences, a fact that could have hindered and/or influenced the perception of hate speech. In addition it is important to acknowledge that the majority of the participants in this study were female. Despite the inclusion of new empirical data, this gender imbalance may introduce a potential bias in the findings and limit the generalizability of the results to a more diverse population. Furthermore, it should also be pointed out that the experimental design of the test in the classroom restricts the extrapolation of the results obtained to those that could have been found in daily life. Therefore, caution should be exercised when interpreting and applying the findings of this study to other contexts and populations.

## 5. Conclusions

Hate speech has received considerable attention in recent years and is increasingly present on the internet and on social networks (cyberhate). However, little research has been carried out to study the perception and identification of such discourse in a sample of young Spaniards. The present study dealt with specific dynamics where the propagation of such discourse was a faithful representation of reality and violent expressions occurring in an unprecedented context: the COVID-19 era.

It would be necessary, based on the research findings, to implement potential intervention strategies aimed at minimizing the impact of such expressions. These strategies could encompass educational and awareness measures to promote tolerance and mutual respect, as well as training programs to identify and address hate speech. Furthermore, conducting future studies to evaluate the effectiveness of these interventions and develop new strategies based on the evolving nature of online hate speech and its social consequences would be crucial. These investigations could contribute to the development of more robust policies and regulations for preventing and combating hate speech, thereby creating more inclusive and secure environments for all individuals.

## Figures and Tables

**Table 1:** Sociodemographic characteristics, personality dimensions, conflict resolution styles and identification with the characters in the study sample.

Variable	*n* = 567
**Age**	21 (20–22)
**Sex**	
Female	413 (72.8%)
Male	153 (27.0%)
Other	1(0.2%)
**Year of study**	
First	109 (19.6%)
Second	184 (33.0%)
Third	209 (37.5%)
Fourth	55 (9.9%)
**Faculty**	
Public	360 (63.5%)
Concerted	207 (36.5%)
**Personality dimensions**	
Extroversion	12 (11–13)
Agreeableness	13 (11–14)
Responsibility	12 (11–13)
Neuroticism	11 (10–12)
Openness/intellect	13 (12–14)
Conflict resolution style	
Cooperative	16 (14–18)
Avoidant	17 (13–19)
Aggressive	12 (12–18)
Identification with the characters	52 (42–60)

Note: Quantitative variables are expressed as median (RIC). Qualitative variables are expressed as frequency (%).

**Table 2:** Experience of a similar situation, perceived aggressiveness and responsibility of the characters in the five simulated scenarios.

Scenarios	1 M (RIC)	2 M (RIC)	3 M (RIC)	4 M (RIC)	5 M (RIC)	Comparison between Scenarios χ^2^_(4) *_	*p*
Experience of a similar situation	3 (2–4) ^a^	2 (1–3) ^b^	2 (1–3) ^c^	3 (2–4) ^a^	2 (1–3) ^b,c^	122.314	0.000
Perceived Aggressiveness	4 (3–4) ^b^	3 (3–4) ^c^	4 (3–5) ^a^	4 (3–4) ^b,c^	4 (3–5) ^a^	203.367	0.000
In favor of the vaccine	4 (2–5) ^b^	4 (3–5) ^b^	3 (2–4) ^a^	4 (3–5) ^b^	3 (2–4) ^a^	67.029	0.000
Against vaccination	2 (1–4) ^a,b^	2 (1–3) ^b^	2 (1–3) ^a,b^	2 (1–3) ^a,b^	2 (1–3) ^a^	24.350	0.000
Spectator				2 (2–3)	2 (2–3)	15.940	0.000
Comparison between characters in each scenario χ^2^_(1) *_	68.432	154.568	132.506	206.660	176.850		
*p*	0.000	0.000	0.000	0.000	0.000		

Note: M = Median, RIC = Interquartile range. a–c: Different letters in the same line indicate significant differences (*p* < 0.05) in the post hoc test. * Numbers in parentheses represent degrees of freedom.

**Table 3:** Spearman correlations between personality factors, conflict management style and identification with the characters and the experience of a similar situation, perception of a similar situation, perceived aggressiveness and responsibility of the characters in each simulated scenario.

	Personality					Type of Conflict to Approach			
	Ext	Agr	Resp	Neuro	Open/Intel	Coop	Avo	Aggres	IP
	Scenario 1								
**VSS**	−0.022	−0.036	−0.015	−0.010	−0.036	0.059	0.051	0.075	0.235 **
**AP**	−0.057	0.713 **	0.043	0.029	0.066	0.223 **	0.230 **	−0.938 **	0.007
**RPFV**	0.015	0.061	0.121 **	0.054	0.128 **	−0.014	−0.004	−0.106 *	0.085 *
**RPCV**	0.005	−0.011	−0.054	−0.003	−0.103 *	0.045	0.022	0.069	−0.061
	**Scenario 2**								
**VSS**	0.025	−0.018	−0.027	0.015	−0.050	0.045	0.033	0.100 *	0.170 **
**AP**	−0.071	0.689 **	0.055	0.067	0.073	0.180 **	0.165 **	−0.874 **	0.154 **
**RPFV**	0.061	0.031	0.061	0.074	0.072	−0.007	0.071	−0.053	0.141 **
**RPCV**	−0.062	0.007	−0.042	−0.002	−0.047	−0.015	−0.080	0.048	−0.122 **
	**Scenario 3**								
**VSS**	0.045	−0.057	−0.028	0.008	−0.056	0.087 *	0.084 *	0.060	0.215 **
**AP**	0.010	0.367 **	0.099 *	0.065	0.105 *	0.008	−0.039	−0.348 **	−0.016
**RPFV**	0.123 *	0.046	0.073	0.046	−0.009	0.063	0.101 *	−0.036	0.120 **
**RPCV**	−0.029	0.003	−0.065	0.006	−0.002	−0.005	−0.088 *	0.064	−0.085 *
	**Scenario 4**								
**VSS**	−0.030	−0.027	−0.004	0.066	−0.014	0.093 *	0.092 *	0.099 *	0.190 **
**AP**	0.062	0.258 **	0.087 *	0.015	0.064	0.054	0.050	−0.314 **	0.108 *
**RPFV**	0.147 **	0.020	0.090 *	0.046	0.072	−0.047	−0.019	−0.041	0.091 *
**RPCV**	−0.058	0.045	0.019	0.041	0.039	0.013	−0.051	0.028	−0.070
**ROP**	0.077	0.019	−0.020	−0.003	0.047	0.031	0.014	−0.020	0.122 **
	**Scenario 5**								
**VSS**	0.030	−0.026	−0.033	0.004	−0.074	0.076	0.078	0.037	0.202 **
**AP**	0.046	0.183 **	0.106 *	0.076	0.095 *	0.011	0.028	−0.214 **	0.053
**RPFV**	0.083 *	0.028	0.046	0.017	0.029	−0.015	−0.030	−0.023	0.030
**RPCV**	−0.014	0.107 *	−0.010	0.067	0.022	0.006	−0.052	−0.030	−0.093 *
**ROP**	0.104 *	0.031	0.055	0.093 *	0.064	0.018	0.023	−0.029	0.092 *

Notes. * *p* ≤ 0.05; ** = Ext = *p* ≤ 0.01; Ext = extroversion; Agr = agreeableness; Resp = responsibility; Neuro = neuroticism; Open/intel = openness/intellect; Coop = cooperative; Avo = avoidant; Aggres = aggressive; IP = identification with the characters; VSS = experience of similar situation; AP = perceived aggressiveness; RPFV = responsibility of the character in favor of vaccination; RPCV = responsability of character antivaccine; ROP = responsibility of the passive observer.

**Table 4:** Spearman correlations between the experience of a similar situation, perceived aggressiveness and character responsibility in each simulated scenario.

	Scenario 1				
	VSS	AP	RPFV	RPCV	
VSS	--				
AP	0.066	--			
RPFV	−0.047	0.095 *	--		
RPCV	−0.022	−0.060	−0.684 **	--	
	**Scenario 2**				
	VSS	AP	RPFV	RPCV	
VSS	--				
AP	0.053	--			
RPFV	−0.083 *	0.097 *	--		
RPCV	0.068	−0.129 **	−0.569 **	--	
	**Scenario 3**				
	VSS	AP	RPFV	RPCV	
VSS	--				
AP	−0.174 **	--			
RPFV	0.074	−0.002	--		
RPCV	0.059	−0.082	−0.258 **	--	
	**Scenario 4**				
	VSS	AP	RPFV	RPCV	ROP
VSS	--				
AP	−0.015	--			
RPFV	−0.122 **	0.096 *	--		
RPCV	0.073	−0.087 *	−0.344 **	--	
	0.139 **	−0.072	−0.242 **	0.120 **	--
	**Scenario 5**				
	VSS	AP	RPFV	RPCV	ROP
VSS	--				
AP	−0.102 *	--			
RPFV	−0.102 *	0.130 **	--		
RPCV	−0.026	−0.091 *	0.048	--	
ROP	0.115 **	−0.188 **	−0.119 **	0.106 *	--

Notes: * *p* ≤ 0.05; ** = Ext = *p* ≤0.01; VSS = experience of similar situation; AP = perceived aggressiveness; RPFV = character with responsibility in favor of vaccination; RPCV = character with antivaccine responsibility; ROP = responsibility of the passive observer.

## Data Availability

The data presented in this study are not publicly available due to privacy restrictions.
